# Disparity of Phoresy in Mesostigmatid Mites upon Their Specific Carrier *Ips typographus* (Coleoptera: Scolytinae)

**DOI:** 10.3390/insects11110771

**Published:** 2020-11-08

**Authors:** Marius Paraschiv, Gabriela Isaia

**Affiliations:** 1 National Institute for Research and Development in Forestry—“Marin Drăcea”, Brașov Station, 13 Cloșca, 500040 Brașov, Romania; marius.paraschiv@icas.ro; 2 Faculty of Silviculture and Forest Engineering, Transilvania University of Brașov, Șirul Beethoven 1, 500123 Braşov, Romania

**Keywords:** *Ips typographus*, phoresy, phoretic mites, Romania, dynamic of phoresy

## Abstract

**Simple Summary:**

This study investigated the phoretic relationship between mites and one of the most aggressive spruce bark beetles from Eurasia. During one season (April–September), bark beetles *Ips typographus* were collected with a specific synthetic aggregation pheromone. In the lab, we investigated the diversity of mites associated with *I. typographus*, mite preferences concerning the body parts of the beetles and how phoretic relationships change during the bark beetle’s flight season. Six phoretic mites species were found and 20% of beetles carried mites. Phoretic mite loads and the percent of beetles with mites were highest during the spring flight period. Phoretic mite species had specific preferences regarding their location on the body of the carrier.

**Abstract:**

*Ips typographus* Linnaeus, 1758, the most important pest of Norway spruce (*Picea abies* Linnaeus, 1753) from Eurasia has damaged, in the last decades, a large area of forest in Romania. Associations between beetles and their symbiotic fungi are well known compared to beetle-mite relationships. The objectives of the study are to determine: (i) the diversity of mites species associated with *I. typographus* in a local outbreak from Central Romania; (ii) the mite’s preferences concerning the body parts of their carriers; and (iii) how phoresy changes during seasonal flight activity of the host. A total of 7896 adult *I. typographus* were analyzed and six mite species (both adults and immature stages) were found: *Dendrolaelaps quadrisetus* Berlese,1920, *Proctolaelaps fiseri* Samsinak, 1960, *Trichouropoda polytricha* Vitzthum, 1923, *Histiostoma piceae* Scheucher, 1957, *Uroobovella ipidis* Vitzthum, 1923, and *Uroobovella vinicolora* Vitzthum, 1926. Most mites were observed under the carriers’ elytra (46.8%), while 26.7% and 25.8% were seen on the thorax and elytral declivities, respectively. Mite phoresy peaked in the spring corresponding to the dispersal flight of the carrier. A smaller peak in phoresy occurred in the summer during the second beetle generation.

## 1. Introduction

Norway spruce (*Picea abies*) is among the most abundant and economically important tree species in Romania and Europe [1,2,3]. These trees are common attacked by bark beetles, the most aggressive of which being *Ips typographus* (Coleoptera: Scolytinae) [4,5,6]. In the Romanian Carpathian forests, *I. typographus* attack together with *Pityogenes chalcographus*, usually having two generations per year, the female being able to resume the attacks several times causing significant damage [5,7,8,9,10]. *Ips typographus* and *P. chalcographus* attack weakened trees [6] where their offspring develop [11]. Adult beetles introduce fungi that alter the wood and, in some cases, provide nutrients for their larvae [12,13]. The spores of most fungi are attached to the body of adult beetles [14], but some fungal species are transported by acarofauna [15], like *Tarsonemus* mites, which have a special structure called a sporotheca [16]. Fungal spores can also stick to mites and are known to stick to the cuticula produced by *Trichouropoda* mites [17]. Some fungivorous mites will actively transport spores to introduce their own food [18].

Mite species are not capable of flight or long-distance travel and, thus, use animals, including bark beetles, to colonize new habitats [19,20]. This form of transport represents an ecological adaptation and is known as phoresy [21,22]. Although not considered a typical parasitic relationship, phoresy can affect the carrier during transport by weighting the carrier down in case of a large number of phoretic mites, affecting bark beetle flight [23,24], albeit controversial [25]. Furthermore, this short-term relationship can, subsequently, have dramatic effects upon the carrier, which may be antagonistic [26,27] or mutualistic [28,29,30,31,32] depending on the mite species vectored. Due to their small size, mites can colonize relatively inaccessible places before other animals [20], and in the case of bark beetles can vector many fungal species, thus creating complex associations and interactions under the bark [33]. Of the more than 1 million terrestrial mites species living on Earth [34], there are around 58,000 described [35,36,37,38], out of which only a small group of less than 270 species are associated with bark beetles [29,32].

The temporary associations between mites and fungi ends when the living conditions are no longer suitable for bark beetles. Mites attach to their carriers by chelicerae, claws, or anal pedicel secreted by the perianal glands [21,39,40]. They utilize external beetle parts like antennae, legs, and wings but some mite species have preferences for specific locations or beetle parts [41]. For a safe dispersal, mites must synchronize their life cycle to one of the carriers, but little is known about how and when mites locate their hosts [42].

Only some of the associations among mites, fungus, and carriers have been studied [16,29,32,33,43]. Relations between mites and nematodes and their carriers have been mentioned in a few studies [44,45]. However, most of the studies have a more faunistic approach and are aimed to emphasize the diversity of mites associated with beetles from a significant part of Europe [46,47,48,49,50,51,52,53,54,55,56,57], including Romania [58,59]. Beetle sampling methods vary across studies and many only focus on one beetle flight period. There are no studies in Romania addressing phoresy dynamics of *I. typographus* populations.

Our goal is to document and understand phoretic mite associations of the most aggressive bark beetle in Romania, *I. typographus*, during its entire flight season. Specifically, our study aimed to determine: (i) the diversity of mite species associated with *I. typographus* in a local outbreak in Central Romania; (ii) phoretic mite preferences concerning the body parts of their carriers; and (iii) how phoresy changes during the seasonal activity of their host.

## 2. Materials and Methods

### 2.1. Study Area

The study area is located in the center of Romania, near the City of Brasov (Brasov County) at an altitude of 975 m.a.s.l. (meters above sea level) in a 120 year-old spruce stands (N45°37′21.00″ E25°32′53.26″). The site consisted of a 3 ha area of forest damaged by windstorm in 2009, enlarged subsequently by an outbreak of *I. typographus.*

### 2.2. Field Sampling and Laboratory Handling

Adults of *I. typographus* were captured using 15 Intercept^®^ traps baited with commercial product AtraTYP^®^ (Raluca Ripan Institute for Research in Chemistry, Cluj-Napoca, Romania). The traps were placed around the perimeter of the outbreak at 12–15 m from the forest edge, keeping 30–35 m distance between them. Lures consisted of a synthetic mixture of cis-Verbenol, Methyil-butenol and Ipsdienol, embedded in a polyethylene bag, with a daily average release rate of about 30 mg/day [60]. Collection of the beetles was made every 7–12 days during 22 weeks, starting when beetles began their flight activity (end of April, 29 April 2011) and finished mid-September, when beetle flight activity ended (mid-September, 12 September 2011).

The air temperature and humidity, used to monitor beetles flight activity, were recorded with data logger devices (HOBO, V23^®^) throughout the field collections and analyzed using dedicated software (HOBOWARE^®^, Onset Company, Bourne, MA, USA).

Captured beetles were identified and 50 living beetles from each trap (15) and collection date (20), were selected for phoretic mite analysis. If there were fewer than 50 captured live beetles, all possible beetles were analyzed [60,61]. Live beetles were placed immediately into −5 °C freezer to prevent mites from leaving their hosts and remain in their original position during phoresy [49]. Because sex determine in *I. typographus* is difficult [62,63], we had to dissect beetles and, using a stereomicroscope, identify the copulatory organ of the male [60]. Prior to beetle sex determination, the number and position (head, thorax, abdomen, legs, etc.) of mites were recorded. Phoretic mites were removed with a pin and cleared in lactophenol solution to be mounted on the microscope slide. Identification was made by using the latest determination keys, and voucher specimens stored at the laboratory of INCDS (National Institute for Research and Development in Forestry)—Brașov. Mites that fell off during storage or in the flight trap cup were not counted.

### 2.3. Data Analyses

Hartley and Shapiro–Wilk tests were used to verify the homogeneity of the distribution for beetle captures and mite phoresy rates. To investigate the effects of the number of mite species identified (six species), data collection (20 weeks), insect sex (two genders) as well as the interaction between pair of fixed factors like the average abundance of mites per insect, the variance was tested with ANOVA, the level of significance established using the Tukey test (Tukey’s multiple test). The mite species *Histiostoma piceae* and *Proctolaelaps fiseri* were excluded from this analysis because of their scarce frequency and numbers. χ^2^ multiple pair-wise comparison tests were performed to identify the preferences of mites for specific body parts of the insects: head, thorax, abdomen, under elytra, and elytral declivity. The level of significance was corrected with Bonferroni sequential in order to adjust the *p*-values by dividing *p*-resulted values to the number of performed tests. All tests were performed using R Statistical Software v.2.15.3 (http://www.R-Project.org).

Species communities were analyzed by the Shannon diversity index (H’), with natural logarithm [64] and its components. The zoocenological analysis of mite communities was based on the indices of dominance (D) and frequency (F), used also, by other authors [50]. Dominance classes were: eudominant (>30%), dominant (15.01–30%), sub-dominant (7.01–15%), resident (3.01–7%) and sub-resident (<3%). The frequency classes were: euconstant (>50%), constant (30.01–50%), subconstant (15.01–30%), accessory species (5.01–15%), and accidental occurrence (<5%).

## 3. Results

### 3.1. Diversity of Mites and Zoocenological Pattern

A total of 58,784 insects were captured in flight traps with an average of 3198.93 insects/trap, from which 7896 bark beetles were examined. Of these bark beetles, 1536 (19.45%) had mites on their body, belonging to six species: *Dendrolaelaps quadrisetus* Berlese, 1920, *Trichouropoda polytricha* Vitzthum, 1923, *Uroobovella ipidis* Vitzthum, 1923, *Uroobovella vinicolora* Vitzthum, 1926, *Histiostoma piceae* Scheucher, 1957, and *Proctolaelaps fiseri* Samsinak, 1960 (Table 1). The phoresy rate was 17.70% for males and 20.18% for female beetles. The Shannon–Wiener diversity index of phoretic species during the whole study period varied from 0.2 (mid-May) to 3.34 (mid-June), with an average value of 1.77. This temporal pattern can be explained by changes that occurred in evenness, which was around 0.51, varying from 0.01 (end of mid-May) to 0.81 (mid-August). Additionally, species mean richness narrowly varies from three to four phoretic species per beetle species along the studied period.

**Figure 1:**
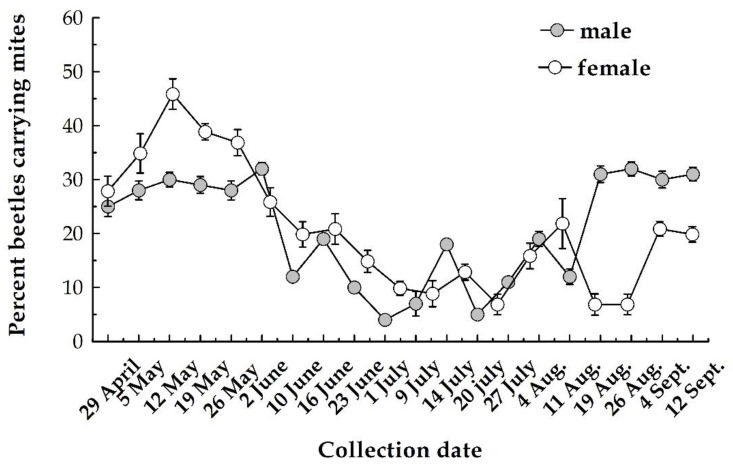
Phoresy of mites on males and females of *I. typographus*.

*Dendrolaelaps quadrisetus* and *T. polytricha* were the most abundant and frequent mite species, together accounting for almost 85% of the total mites identified (Table 1). The less abundant (subresidual) and frequent (accidental) species were *H. piceae*, *U. vinicolora* and *P. fiseri*. Phoresy rates indicate a significant effect of collection date (df = 19, f = 2.658, *p* < 0.001), but no effect of phoretic mites on male and female beetles (df = 1, f = 1.367, *p* = 0.740), even though some differences were noticed at the end of the beetle flight season (Figure 1).

Of all of collected beetles with mites, 85.5% of beetles had one mite species, 13.5% had two mite species, and 1% carried 3 species. For beetles with only one species, 43.2% had (only) *D. quadrisetus*; 28.4% had *T. polytricha*; 13.4% had one of the two Uroobovella species and 0.5% had *H. piceae*.

The most frequent phoretic mite combinations were those of *D. quadrisetus* and *T. polytricha* (6.4% of beetles with mites), and T. *polytricha* and *U. ipidis* (4.1% of beetles with mites).

### 3.2. Location of Phoretic Mites on the Beetles’ Body

Most phoretic mites were located under the beetles’ elytra. Other locations, such as the thorax (prothorax and metathorax), recorded 26.7% of the mites, the elytral declivity had 25.8% of mites, whilst the smallest number of mites, 0.7%, was from the ventral abdomen (Table 2).

Over 99% of *D. quadrisetus* were located under the elytra (Bonf. corr. *p* = 0.018), while *T. polytricha* was more often seen on the thorax (53%) and the elytral declivity (45%), although not significant (Bonf. corr. *p* = 0.00922). The species of *Uroobovella* were primarily located on the elytral declivity (59.7%) and thorax (39.2%), with significant preferences between those (Bonf. corr. *p* = 0.0134). *H. piceae* showed a significant preference (Bonf. corr. *p* = 0.027) for under the elytra, while *P. fiseri* were primarily found outside of the elytra (Bonf. corr. *p* = 0.032).

Male and female beetles did not significantly differ concerning the position occupied by mites over time (df = 1, f = 1.129, *p* = 0.786).

### 3.3. Carrier Activity and Dynamics of Phoresy

#### 3.3.1. *Ips typographus* Flight Activity

Beetle flight activity started when day temperatures (maximum temp. around noon) of 16.5° were recorded, between 23 and 29 April, when the average capture was 6.13 ± 1.9 beetles per trap. The activity increased until 5 May (25.3 ± 6.8 beetles/trap) and, due to unfavorable conditions (temperature below 16.5 °C and rainy days), reduced to 1.4 beetles/trap. After that, the number of beetles increased considerably until mid-June when 757.3 ± 74.8 beetles/trap were recorded. At this date, we can consider that first flight period, due to the emerging of the overwintering parents (P), ended. After this date, the development of the first generation of the beetles (F1) began, as well as sister broods (S) which was marked with lower catches, up to 145.3 ± 21.6 insects/trap.

The entire development of the first-generation (F1) is marked by low captures, the turning point being recorded at the beginning of July, with captures of 334.46 ± 48.5 insects/trap, which represent the intensification of the activity of the adults (those that have wintered) and will complete the sister brood (S).

The third flight period was recorded between mid-July to September. This last part of the season (F2) is characterized by low captures, from 2.13 ± 0.6 to 152 ± 18.2 insect/trap, with a total of 9486 beetles, 1.53 times lower than the second period (F1), and 3.66 times lower than the first interval (P).

Overall, the overwintering adults (P) represented 59.11%, the first generation 24.75% (F1), and the second one (F2) 16.14% of the total captured beetles.

Male beetle numbers were relatively higher (57.1%) at the beginning of the flight and continually decreases until the end of May (38.4% males). Percentages of male beetles were not higher than 35.3%, except during the sister brood, when the male percentage was around 40%. Differences in the relative abundance of the two sexes were significant different (df = 19, f = 3.750, *p* < 0.0001). There were no differences between beetle traps (df = 14, f = 1.210, *p* = 0.328).

#### 3.3.2. Dynamics of Phoresy

**Figure 2:**
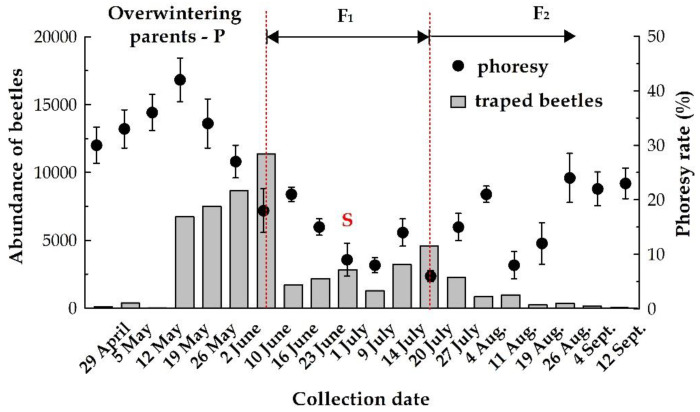
Dynamics of phoresy rate during *I. typographus* activity. End of May-beginning of June (first peak): emerging of overwintering parents (P) and beginning of the first generation (F1); June (second peak): reemergence of P and beginning of sister generations (S); July (third peak): swarming of F1 offspring and beginning of the second generation (F2).

Phoretic rates varied over time (df = 19, f = 5.962, *p* < 0.0001) but there were no significant differences between all 15 traps (df = 14, f = 0.768, *p* = 0.710). Phoretic rates oscillated between 6.4% and 42.1% (beetles with mites) with two peaks, spring and summer (Figure 2). The beginning of May coincided with a phoresy rate of 33.1% (Figure 2) and increased to a rate of 42.1% by mid-May. Phoresy rates then decreased continuously, reaching in July values between 6.4% and 14.3%. After 20 July, the percentage of beetles with phoretic mites gradual increased as beetle trap catches decreased.

The analysis indicates a significant change in phoretic load during the sampling periods (df = 3, f = 3.850, *p* < 0.001), but insignificant in terms of host sex (df = 1, f = 1.864, *p* = 0.740).

**Figure 3:**
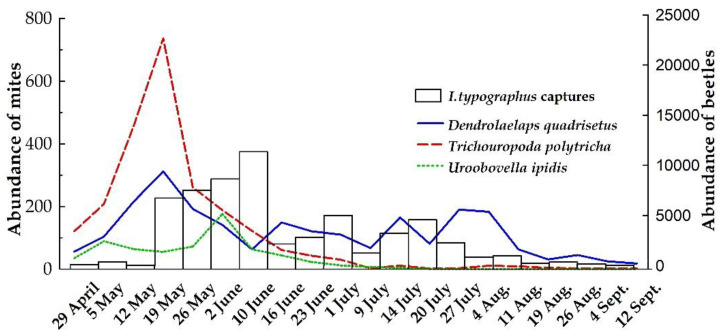
Abundance of mite species during seasonal flight activity of the carrier. Left axis—number of mites recorded, and right axis—number of trapped beetles.

For *D. quadrisetus*, phoretic load varied between 0.09 and 0.92 mites/insect, with the highest peak in 19 May (Figure 3). Frequently, 1–3 individuals of this species (2.23 ± 1.97 mites/beetle) were seen on *I. typographus*, but one male had 26 *D. quadrisetus* under elytra.

*T. polytricha* ranged from 1.0 to 4.47 mites/beetle with the maximum phoretic loads on 19 May.

Most of *U. ipidis* and *U. vinicolora* (80.2%) appeared during May and June. Unlike *T. polytricha*, the maximum phoretic loads of *U. ipidis* were recorded at the beginning of July, with 0.52 mites/beetle, after which it appears sporadically. *U. ipidis* fluctuated between 1.0 and 3.12 mites/beetle with an average value of 1.64 ± 1.38 mites/beetle. Additionally, all 34 *U. vinicolora* mites were collected from May to June. *U. ipidis* fluctuated between 1.0 and 3.12 mites/beetle with an average value of 1.64 ± 1.38 mites/beetle.

The small number of *H. piceae* specimens (14 individuals) collected at the beginning of June and July did not allow detailed analysis of phoretic interaction of this species, while for *P. fiseri* most of the individuals were collected between the beginning of May and the end of June.

**Figure 4:**
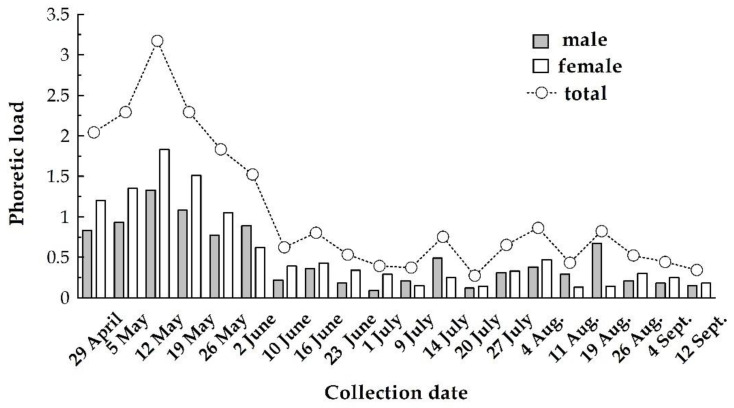
Phoretic load on both sexes of *I. typographus* (average mites/beetle)**.**

The abundance of mites on male and female beetles varied slightly during the season (Figure 4), from a minimum of 0.36 mites/beetle at the beginning of July to a maximum of 3.16 mites/beetle in mid-May.

## 4. Discussion

### 4.1. Diversity and Zoocenological Pattern

The phoresy rates recorded in this study (~5–45%) varied in time but were within the range of phoretic rates found in Southern Germany [49] (30–36%), Poland (30%) [50], and Sweden (23%) [46]. The significant variation in the percentage of beetles carrying mites in our study likely results from the long sampling period (i.e., throughout flight season) of 22 weeks, a period in which two generations of beetles developed.

The small number of mite species found in this study is in agreement with some authors who discovered six species of mites on *I. typographus* in Poland [50], five species in Bulgaria [55], and eight species on the same insect in the Czech Republic [56], but represents far less than other studies that revealed between 13–25 mite species on the same host [46,47,49,65]. However, it seems that mite communities differ significantly in Europe and the small number of phoretic mite species may be due to local specificity [50] and sampling length. On the other hand, the reduced diversity can be explained by the excessively large number of mites belonging to *D. quadrisetus* and *T. polytricha*. Regardless of the number of phoretic species mentioned in the literature, phoretic mites represent only a small part of the 68 species observed inside the galleries of *I. typographus* [66].

Two of phoretic mite species (*D. quadrisetus* and *P. fiseri*) are believed to be predators. *D. quadrisetus* have also been reported to consume larvae of bark beetles and nematodes, having a constant presence during the development of the first and second generations of *I. typographus*, and consuming 9.7% of bark beetles eggs [27,67]. *D. quadrisetus* has a broad phoretic host range as it has been collected on *I. typographus* [49,55] and other European bark beetle species: *Polygraphus polygraphus*, *Pityogenes chalcographus* or *Pityokteines curvidens* [68,69,70] as well as on the North American bark beetles *Ips pini* [32,71]. *P. fiseri* has also been found on at least 25 species of insects [72], among the following bark beetles: *Dendroctonus frontalis*, *D. tenebrans*, *D. valens*, *Hylurgops palliatus*, *I. avulsus*, *I. grandicolis*, *I. calligraphus*, and *Hylastes sp*. [32].

*T. polytricha* feeding behavior is unknown and has also been found on *I. typographus* in other studies [49,50,55] and other beetles: *I. sexdentatus* [42], *I. duplicatus* [57], *Hylastes cunicularius* [15], *Dendroctonus*, and *Polygraphus* species [32].

*H. piceae* prefers fungal-rich habitats with a high amount of spores and mycelium [29] and can also feed on bacteria and yeasts [17]. In addition to *I. typographus* [49,55], this mite is reported on other bark beetle species: *I. typographus japonicus* [73], *I. cembrae* [74], *P. chalcographus* [69], and *Pityokteines ssp*. [32,54,70]. Furthermore, some authors [69] found that this species may become hyperphoretic on other mite species of (e.g., *U. ipidis*) carried by *P. chalcographus*.

### 4.2. Attachment Places

Houck and O‘Connor [21] hypothesize that the distribution of mites on the host body is nonrandom, with mite species choosing specific parts of the host body during phoresy. Our phoretic data on host location preferences differ considerably from similar work [46], which located 82.5% of mites under elytra, 10.3% on the thorax, 6.5% on elytral declivity, and less than 1% on other parts of the body. Other studies using pheromone traps [49] found 61.5% of the mites under elytra, 24.3% on the thorax, 10.5% on the elytral declivity, and 3.7% on other parts of the body, which shows a similar pattern of preferences for attachment locations. Differences across studies could result from how beetles were stored (alcohol vs. frozen) as well as host population dynamics, mite species composition, and time of season. Even so, the attachment sites are chosen by the mites to avoid being brushed off by the host [75] or to avoid injury during beetle gallery excavation [76]. Differences in attachment sites could also be due to the space available on the beetle at the time of locating and connecting to the insect. Based on our observations, the abdomen of the beetles is not a good place to attach. In any case, the ontogenetic instar of mites—mainly deutonymphs, in our study—influences the attachment preferences since this stage is critical and reduces vulnerabilities during transportation.

Our findings related to the presence of deutonymphs of *D. quadrisetus* attached to the internal part of the elytra confirms other studies that noticed a particular preference for this body part [46,49] even on a different beetle host species [42]. The attachment location for *H. piceae* supports previous findings [46] that indicated the same position on the same host, *I. typographus*, for all mite specimens belonging to this genus. These findings are somehow surprising since this species was shown to exploit some peculiarity in host morphology of *I. typographus* and *I. cembrae*—the mite is observed to “sit “on the host instead of “attaching” [74].

### 4.3. Dynamics of Phoresy

Mites and their potential beetle host must meet each other “at the same time and space” [77] when the beetles are exiting the tree, traveling and entering a new host. As a consequence of this, the existence of phoresy is marked by four crucial moments: (i) initial attachment; (ii) transport to new habitats; (iii) detaching, and resuming the development cycles (for both organisms); and (iv) reattaching to abandon these places after habitat deterioration. For some mite species, diapause is synchronized with beetle developmental stages [78], and maybe this is why in our study the higher quantity of mites was collected from the overwintering parents within the dispersal flight which occurs in early spring. Unlike phoretic mites associated with vertebrate carrion, who have a delay in peak abundances and richness relative to beetle (or host) assemblage [79], in our study the mites reached the peak abundance before peak beetle emergence.

Regardless of the developmental stage of the host, a temporal specificity was observed for all mite species. Even so, the sampling date of the beetles alone could not entirely explain the inconsistencies between our results and previous studies; thus, combined with the “historical status” of the site with respect upon the age of the outbreaks and new sites where the first phoretic loads are smaller than the old ones [80], may give only part of the answer. Another factor that could influence the dynamics of phoretic loads is the seasonal dynamics of their host beetle and their relative densities compared to those of mites within the bark. Studies have found that climate forces the majority of beetle to overwinter in the litter [81], where the probability of the phoretic mite population to growth or survival is small. In our study, we found a higher phoresy rates on beetle populations that overwinter under the spruce bark [82], where mite survival and connection with beetles is much higher.

Morphological instar of the mites, mostly deutonymphs in our study, could be a reason for high peak abundance early in the season, as some species phoresy can only be phoretic during this stage [49]. This is why mites must complete the development when carriers have already finished mating and are creating their maternal galleries, which can last several weeks.

The constancy with which *D. quadrisetus* was found throughout the host’s activity gives this relationship a more intimate character, which would mean more than just a transport relationship (such as a predator-prey association); while the presence of *T. polytricha* and *Uroobovella* sp. indicates an exclusive transport relationship, this being mainly associated during the emerging of the overwintering parents (P) and less during the development of the two generations of beetle progeny (F1 and F2). However, in the case of the *T. polytricha* and both *Uroobovella* species, the issue regarding the timing of their attachment to beetles is not fully clarified since these species practically disappear by the end of the first generation (F1) and are expected to appear only the following spring. Nevertheless, the high rates of phoresy recorded at the beginning of May indicate that this is the time when the massive attachment of the mites to emerging beetles takes place, and this may be the moment when the mite phoretic stage is most active. Not having data for one autumn and the following spring, we cannot state in our study what causes the difference between the recorded rates. We can only speculate on several factors: the age of the outbreak, the local specificity regarding the quantity and the quality of the resources, the mortality but also the proportion between egg-larvae-pupae and the adults that wintered, all of them potentially influencing the abundance and diversity of the mite species between the cessation of the host’s activity within the tree and its emergence the following spring. It was also demonstrated that during the host flight activity, mites could fall from the beetle’s body proving the existence of an indirect association between the amount of the mites and flight distances made by the host [80], which represent the distance from an existing outbreak to new sites.

## 5. Conclusions

In this study, we found six species associated with *I. typographus* population, throughout a flight season. Most mites were located under the elytra’s host or on the thorax and elytral declivity. The greatest phoretic loads occurred on overwintering beetles during early spring. Unlike *D. quadrisetus* which was very frequent during the whole season and are predators of nematodes and bark beetle eggs and larvae, *T. polytricha* and *U. ipidis* were uncommon and may only associate with beetles for transport. We observed no difference between male and female beetle hosts concerning phoresy rates, although some variation in phoresy rates were noticed mainly during the first and second flights (F1 and F2).

Future studies should monitor phoretic mite loads over multiple years and beetle generations to better understand mite population dynamics and how they may affect bark beetle ecology. Studies should also investigate the potential of predatory mite species like *D. quadrisetus* which was demonstrated in other studies to consume beetles offspring, for biological control.

## Figures and Tables

**Table 1:** Phoretic mites associated with *Ips typographus*, their dominance, and frequency.

Family	Species	Number of Phoretic Mites	Dominance (%)	Frequency ^a^ (%)
*Digamasellidae*	*Dendrolaelaps quadrisetus*	1896	45.38 Eudominant	84.18 Euconstant
*Ascidae*	*Proctolaelaps fiseri*	59	1.41 Subresident	12.55 Accesory
*Trematuridae*	*Trychouropoda polytricha*	1652	39.54 Eudominant	53.02 Euconstant
*Histiomatidae*	*Histiostoma piceae*	14	0.34 Subresident	6.51 Accessory
*Urodinychidae*	*Uroobovella ipidis*	523	12.52 Dominant	37.20 Constant
*Urodinychidae*	*Uroobovella vinicolora*	34	0.81 Subresident	3.72 Accidental
	Total	4178	100	19.45

^a^ Classification according to Gwiazdowicz et al. [50].

**Table 2:** Distribution of mites on the bodies of *Ips typographus* beetles.

9Species	Developmental Stage	Under Elytra	Thorax	Elytral Declivity	Abdomen	Total
*Dendrolaelaps quadrisetus*	deutonymphs adults	1878	13	5	-	1896
*Proctolaelaps fiseri*	adults	59	-	-	-	59
*Trychouropoda polytricha*	deutonymphs	5	880	743	24	1652
*Histiostoma piceae*	deutonymphs *	14	-	-	-	14
*Uroobovella ipidis*	deutonymphs	-	205	313	5	523
*Uroobovella vinicolora*	deutonymphs	-	18	16	-	34
Total		1956	1116	1077	29	4178

* hypopus.
